# The effects of reputation on inequality in network cooperation games

**DOI:** 10.1098/rstb.2020.0299

**Published:** 2021-11-22

**Authors:** Milena Tsvetkova

**Affiliations:** Department of Methodology, London School of Economics and Political Science, Houghton Street, London WC2A 2AE, UK

**Keywords:** reputation, cooperation, inequality, networks, experiments, agent-based model

## Abstract

In the last several decades, ample evidence from across evolutionary biology, behavioural economics and econophysics has solidified our knowledge that reputation can promote cooperation across different contexts and environments. Higher levels of cooperation entail higher final payoffs on average, but how are these payoffs distributed among individuals? This study investigates how public and objective reputational information affects payoff inequality in repeated social dilemma interactions in large groups. I consider two aspects of inequality: excessive dispersion of final payoffs and diminished correspondence between final payoff and cooperative behaviour. I use a simple heuristics-based agent model to demonstrate that reputational information does not always increase the dispersion of final payoffs in strategically updated networks, and actually decreases it in randomly rewired networks. More importantly, reputational information almost always improves the correspondence between final payoffs and cooperative behaviour. I analyse empirical data from nine experiments of the repeated Trust, Helping, Prisoner's Dilemma and Public Good games in networks of ten or more individuals to provide partial support for the predictions. Our research suggests that reputational information not only improves cooperation but may also reduce inequality.

This article is part of the theme issue ‘The language of cooperation: reputation and honest signalling'.

## 1. Introduction

We humans have the distinctive ability to form complex social interaction and communication networks. These networks allow us to share information and cooperate with each other, achieving more collectively than any single individual could by themselves. In fact, scientists argue that our ability to gather and disseminate information about others is one of the reasons why we trust and help each other: that is, reputational information promotes cooperation.

Reputation is the information about someone's past behaviour that we obtain from direct observation, from centralized institutions (reputation systems), or via social networks (gossip). In social dilemma situations where cooperation is individually undesirable but collectively beneficial, reputation allows cooperation to emerge via indirect reciprocity or reputation-based partner choice [1]. Indirect reciprocity is the tendency to help individuals who have been helpful towards others, while reputation-based partner choice is the tendency to select helpful partners and avoid unhelpful ones. Indirect reciprocity is evolutionarily advantageous and thus deeply ingrained in us [2,3]. Owing to indirect reciprocity, individuals realize that their decisions affect their reputation, which in turn affects how others treat them in future interactions, and hence they become more likely to cooperate [4–6]. In contrast, owing to reputation-based partner choice, individuals increase their cooperation so that they are more likely to be chosen as partners and benefit from future interactions [1].

By fostering cooperation, reputation increases individuals' final payoffs on average and improves collective wealth. However, how are the payoffs distributed among different individuals? And do they reward cooperators more than defectors? These are essentially questions about inequality. The problem of inequality is one of the most fundamental problems modern organizations and societies face. Inequality decreases individual productivity and job satisfaction at the workplace, lowers organizational performance [7,8], and more generally, negatively impacts happiness [9,10] and health [11]. Thus, a more comprehensive understanding of the effects of reputation systems on inequality would allow us to evaluate any potential tradeoffs and design more efficacious and sustainable organizations, institutions, online marketplaces and social media communities.

This study takes a major step in this direction by investigating the effect of reputational information on inequality in repeated social dilemmas in networks with different dynamics. I investigate inequality in terms of the *dispersion* of final payoffs and the *correspondence* of final payoffs to cooperative behaviour. Looking through the lens of the coevolution of reputations, social networks and cooperation [12], I compare situations where individuals are randomly matched with new partners, such as speed-dating events, round-robin tournaments, anonymous chat rooms, or rotating teams for school assignments and work projects, with situations where individuals can choose with whom they interact, such as online markets or self-assembled project teams. I argue that, when the networks are dictated by random matching, the higher levels of cooperation that reputation produces lower the dispersion of payoffs. However, when the networks are updated strategically, reputation fosters the clustering of cooperators and exclusion of defectors and thus may increase the dispersion of payoffs, yet reduce inequality in terms of better correspondence between payoffs and cooperative behaviour. I use an agent-based model to visualize the group-level expectations and data from nine network cooperation experiments to provide empirical evidence for them.

## 2. Theoretical background

In non-cooperation settings, reputation has long been understood as a prominent mechanism for inequality. If a person's past behaviour and performance are used to predict their future actions and achievements, reputation can produce a positive feedback loop whereby past accomplishments and resources turn into new accomplishments and resources. The process can cause initially small or accidental differences in behaviour and performance to compound and amplify over time. As a result, individual outcomes become less predictable and more extremely distributed. Merton studied this process in the context of academic success and famously labelled it ‘the Mathew effect' [13]. The process is also known as ‘the rich get richer', increasing returns, and cumulative advantage [14,15].

Reputation has been shown to create inequality in cooperation settings too. Hackel & Zaki [16] demonstrate that reputation serves to propagate existing inequalities in cooperation experiments. They find that participants tend to misattribute good reputation to those who have an arbitrary resource advantage that allows them to give more to others. As others rely on this reputational information to make investment decisions, they end up reproducing and reinforcing the inequalities in a different setting. Furthermore, Frey & van de Rijt [17] provide evidence for reputation-driven cumulative advantage in experimental buyer–seller markets. The authors find that sellers who cooperate early get disproportionately rewarded because buyers are more likely to choose sellers of good repute. This implies that initial differences in cooperative behaviour could have long-lasting effects that get reinforced and exaggerated over time. As a result, social groups where information about past behaviour is available will have higher inequality than groups without reputation tracking.

Evaluating inequality in the context of cooperation, however, is a complex problem. Cooperative behaviour is collectively beneficial and hence valued and desirable. Importantly, cooperative behaviour is not necessarily inherent but conditional on others. If we disassociate individuals from the cooperative behaviour they exhibit, then we can measure inequality with the dispersion of final payoffs. In essence, if we consider differences in cooperative behaviours emergent, and hence, somewhat arbitrary, then a higher payoff difference between the wealthiest and the poorest would define higher inequality. On the other hand, however, if we hold individuals accountable for their cooperative behaviour, then inequality will depend on the extent to which payoffs correspond to cooperation. If we assume defectors willingly choose to defect, then situations where they end up better off compared with cooperators would be more unequal. This distinction between *dispersion* and *correspondence* parallels the contrast between dispersion and rank reversal that Freda Lynn and colleagues delineate in the context of status hierarchies [18]. The underlying idea is that if we start with a population that is heterogeneous on a valued characteristic or behaviour, then inequality can take two forms: outcomes that are more dispersed than the underlying heterogeneity, and outcomes that correspond less to the valued characteristic or behaviour.

Furthermore, in cooperation settings, reputation can have complex effects on inequality. On the one hand, we know that reputation tends to increase the level of cooperation. Starting from the initial rounds of interaction, individuals cooperate more owing to forward-looking behaviour: aware that their reputation will affect others' behaviour towards them in the future, they immediately start investing in it [4,19]. Over the course of interacting, individuals further learn from experience: they realize that being a reputable partner gives one an advantage and consequently, switch to cooperating [20]. Since more uniform behaviour entails more similar individual outcomes, the higher levels of cooperation that reputation brings can lower the dispersion of final payoffs. Multiple studies confirm that reputation promotes cooperation in simple dyadic interactions [21–23], but there are also studies that fail to find the expected effect [24].

Higher levels of cooperation will entail lower payoff dispersion if there is no segregation between cooperators and defectors. However, if reputation allows cooperators to find other cooperators and exclude defectors, the differences in payoffs between the two groups can widen. Simultaneously, the correspondence between payoffs and cooperative behaviour will improve. Indeed, previous research shows that individuals are more likely to select partners who have reputation as cooperators [17], as well as more likely to cooperate with them [23,25]. At the group level, this implies that reputation will lead to networks with higher clustering by cooperativeness and higher degree centrality for cooperators. In fact, network experiments where payoff depends on the number of partners show the emergence of cooperative hubs as cooperative individuals attract higher numbers of connections [17,19]. The evidence for clustering by cooperativeness, however, is less convincing. Melamed *et al*. [26] find no effect from reputation on clustering as partner choice alone can induce near-uniform cooperation. Gallo & Yan [19] discover that reputational information needs to be combined with knowledge of the network in order for cooperators to cluster.

One prior study that uses data from nine network cooperation experiments to investigate the effects of reputational information on the dispersion of final payoffs reports statistically significant effects in both the positive and negative directions, as well as near-zero effects [27]. Here, I extend this work in a couple of ways. First, I present a more nuanced view of inequality by investigating both the dispersion of payoffs and the correspondence between payoffs and cooperative behaviour. Second, I pinpoint one specific factor that moderates the effect of reputational information on inequality: the dynamics of the underlying interaction network. I compare the effects of reputation under homogeneous mixing in randomly rewired networks with those under strategic partner selection in dynamic networks.

## 3. Expectations

I use an agent-based model to demonstrate how the effects of reputational information on payoff dispersion and correspondence may differ for different network dynamics [28]. I rely on a generic and simple model that assumes agents follow fixed behavioural strategies and heuristic-based rules to respond to others' actions and reputational information. The model I use intentionally avoids problematizing the evolution of cooperation (in contrast to most prior work) and instead takes it for granted in order to focus on how cooperation affects the distribution of payoffs. My goal is to demonstrate the theoretical complexity of the phenomenon, rather than provide quantitative predictions for the empirical analyses.

The agents in the model play an *N*-person Prisoner's Dilemma game in which they choose between cooperating, C, and defecting, D, with payoffs for mutual cooperation CC = 5, mutual defection DD = 2, defecting with a cooperating partner DC = 8, and cooperating with a defecting partner CD = 0. Following empirical research on behavioural proclivities in the general population [29,30], I assume that agents belong to three different fixed-strategy types: fraction *p*_D_ are persistent defectors, *p*_A_ are persistent altruists, and *p_R_* = 1 − *p*_A_ − *p*_D_ are conditional cooperators. Altruists always cooperate, defectors never do, and conditional cooperators initially cooperate with some probability, after which they reciprocate by cooperating if at least a certain fraction of their interaction partners cooperated in the last period. While *p*_A_ and *p*_D_ define the minimum and maximum possible levels of cooperation, respectively, *p_R_* determines the proportion of responsive and interdependent agents, who exhibit more realistic variation in behaviour and drive the system's emergent properties.

Agents interact in a network of size *N* and average node degree *m*. To compare the effect of network dynamics, I study two modes of network updating. For the randomly rewired networks, agents are placed in a new network every period. For the strategically updated networks, every period each agent is given the opportunity to replace one of their defecting neighbours with someone else. An existing link gets deleted if one of the two agents drops it, but for a new link to appear, both agents need to desire it. To facilitate mutual nominations but avoid skewed degree distributions, I assume that agents can nominate multiple new potential partners, as long as the number of actual partners does not exceed 2*m*.

To avoid stochastically unstable outcomes, I assume a small probability for error *ε* = 0.005 such that the agent executes an action that is opposite to the one they originally chose, and another small probability for error *γ* = 0.005 such that the agent does not update their network even if they have decided to.

I study the outcomes in networks with random rewiring and strategic updating when there is no reputational information and when reputation is provided as the average action over the last *r* periods. When reputational information is available, forward-looking individuals are more likely to cooperate when they are aware of the negative consequences from reputation as a non-cooperator [4,19,26]. I implement this by specifying that conditional cooperators initially cooperate with probability *θ*_0_ + *a*_0_*r*, and then cooperate if at least *θ*_C_ − *a*_C_*r* of their neighbours cooperated over the last *r* periods, where *θ*_0_ and *θ_C_* are the behavioural thresholds without reputation and *a*_0_ and *a_C_* define the strength of the reputation effects (0 < *θ*_0_, *θ*_C_, *a*_0_, *a*_C_ < 1). Reputational information also comes in play when agents select new partners [17,19,26]. Without reputational information, agents pick new partners randomly from those with whom they are not yet linked. Otherwise, they pick only among those who have cooperated in at least *θ_C_* of the past *r* periods.

For the results I report here, I fix the initial tendency to cooperate *θ*_0_ = *p*_A_, that is, I assume that the willingness to cooperate on first encounter is equal to the probability of encountering an unconditional cooperator. Additionally, I fix the subsequent cooperation threshold *θ_C_* = 0.5 and the reputation effects *a*_0_ = 0.1 and *a_C_* = 0.05. Generally, higher *θ*_0_, *p*_A_, *a*_0_, *a_C_* and lower *θ_C_* increase the rate at which cooperation emerges and the equilibrium level of cooperation. The specific values are chosen because they produce sufficient variation in the level of cooperation and replicate the empirically validated expectation that reputational information and strategic network updating independently increase the level of cooperation (electronic supplementary material, figures S1A, S2A, S3A). Furthermore, I assume that agents are initially embedded in a random network, where an interaction link between any pair of nodes is formed with a small fixed probability; this produces initial networks with low clustering and Poisson degree distributions, where I fix the network size *N* = 100 and average node degree *m* = 2. I also assume that payoffs are averaged over all interactions in a period and, hence, do not depend on the number of interaction partners. The model allows modification of the assumptions about the initial network structure, average node degree *m* (see electronic supplementary material, figure S1), payoff function (electronic supplementary material, figure S2), and reputational information *r* (electronic supplementary material, figure S3), but the outcomes are qualitatively similar.

Although the model is simple enough to be solved analytically, I focus here on visualizing the theoretical intuition and demonstrating the variability of outcomes expected for a range of cooperation outcomes due to changes in the proportion of persistent altruists, persistent defectors, and conditional cooperators. I measure inequality in terms of dispersion with the Gini coefficient of the payoffs accumulated after *T* = 100 periods. The Gini coefficient is 0 when all individuals have equal payoffs and 1 when only a single individual has a non-zero payoff. I measure inequality in terms of correspondence with the Pearson correlation between the proportion of periods in which the agent cooperated and the agent's final payoff. A Pearson correlation of 1 means that higher cooperation is always proportionally rewarded with higher payoffs, while a Pearson correlation of –1 indicates perfect inverse proportionality.

**Figure 1.**
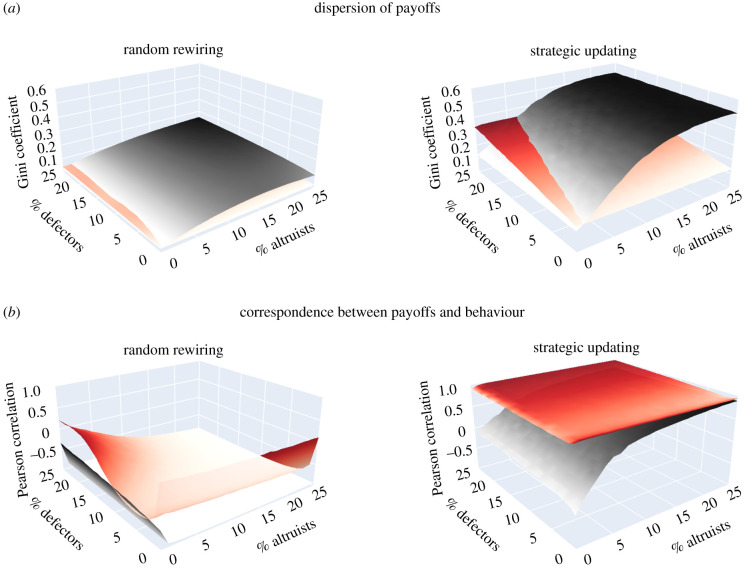
(*a*) Agent-based models reveal that reputation (shown in red) generally decreases the dispersion of payoffs in randomly rewired networks but might increase in networks with strategic updating when the percentage of steady defectors is sufficiently high. (*b*) Reputation almost always results in better correspondence between payoffs and cooperative behaviour. The plots show (*a*) the mean Gini coefficient of accumulated payoffs and (*b*) the mean Pearson correlation between proportion of periods cooperating and final payoff when no reputational information is available (*r* = 0; grey) and when agents know everyone's actions in the previous period (*r* = 1; red). The means are calculated over 1000 runs in networks of *N* = 100 agents who start interaction in a random network with *m* = 2 partners on average and play for *T* = 100 periods. The simulations vary the per cent of steady defectors (*x*-axis) and steady altruists (*y*-axis), with the rest being conditional cooperators.

The model comparing outcomes for *r* = 0 and *r* = 1 confirms the intuitive expectation (figure 1). The results indicate that reputational information decreases the dispersion of payoffs in randomly rewired networks but could increase it in strategically updated networks when persistent defectors are numerous (figure 1*a*). In randomly rewired networks, the Gini coefficient is always low but reputation brings it even lower because conditional cooperators can make agents pay back for last-period's defection. In strategically updated networks, the Gini coefficient increases with higher proportions of persistent altruists in the absence of reputation but with higher proportions of persistent defectors when reputational information is available. The reason is that reputation allows the complete isolation of defectors, while its lack does not prevent the exploitation of altruists.

Although reputational information can increase the dispersion of payoffs in strategically updated networks under certain conditions, it almost always provides better correspondence between payoffs and cooperative behaviour, regardless of the network dynamics (figure 1*b*). With strategic updating, reputation guarantees that defectors are excluded and thus suffer, while cooperators prosper. Under random rewiring, even though reputation rarely produces positive rewards for cooperation, it still reduces the rewards for defection by making payoffs less negatively correlated with cooperative behaviour. In short, reputational information combined with the strategic choice of partners could increase the dispersion of payoffs, but this is because persistent defectors deservingly lose out.

## 4. Data and methods

I provide empirical evidence for the theoretical expectations with data from nine network cooperation experiments (table 1). These experiments were originally designed to study the effect of reputational information on the emergence of cooperation and the published articles associated with them do not report on inequality. I identified these studies after conducting online searches on the Scopus database, the Google Scholar search engine and the Cooperation Databank [31]. Specifically, I searched for network cooperation experiments that involve the repeated play of a social dilemma game in networks of at least 10 and that manipulate the information available on other participants' past actions. I only considered accurate and objective information, excluding studies with gossip or subjective ratings. I identified 10 studies and, after contacting the corresponding authors, obtained data for nine of them (with [19] missing) [28].

**Table 1.** Summary of the experimental data. The games played in the experiments are Prisoner's Dilemma (PD), targeted Prisoner's Dilemma (tPD), Public Good (PG), Helping (HG) and Trust (TG) games. Payoffs are shown for *CC*, *CD*, *DC*, *DD* for PD and *CC*, *CD*, D for TG. For HG, the payoff numbers correspond to *cost*, *benefit* for the gift, and for PG, *c_i_* (*c_j_*) is the amount contributed by the player (all players) to the public good. *N_i_* is the number of unique participants, *N_*n*_*—number of networks, *N*—network size, *T*—number of periods. Network refers to network structure, *m*—the number of interaction partners, and updating—how the network is updated. Values in brackets refer to the network in the first period only. Reputation refers to reputation tracking, with the number indicating the number of previous periods over which the partner's actions are observed; all—observe all actions in previous periods; avg—observe average behaviour over all previous periods; last—observe average behaviour over previous four-period-long phase; part—observe average behaviour over 50% of previous periods; loc—observe average behaviour for partners' partners only; 1 + 1—observe partner's action in last period, as well as the action of the partner's partner from the period before that.

experim.	game: payoffs	*N_i_*	*N_*n*_*	*N*	*T*	network	*m*	updating	reputation
BOLT04	TG: 50, 70, 35	144	9	16	30	pair	1	random	0, all
BOLT05a	HG: −0.25, 1.25	96	6	16	14	pair	1	random	0, 1, 1 + 1
BOLT05b	HG: −0.75, 1.25	96	6	16	14	pair	1	random	0, 1, 1 + 1
SEIN06	HG: −150, 250	112	8	14	>90	pair	1	random	1, 6
STAH13	PD: 80, 10, 90, 20	92	8	22, 24	24, 39	pair	1	random	0, 1
BAYE16a	PG: 100 – *c_i_* + 0.8Σ*c_i_*	224	12	16–20	24	pair	1	random	0, last
CUES15	PD: 7, 0, 10, 0	243	22	17–25	25	(cycle)	(4)	strategic	0, 1, 3, 5
BAYE16b	PG: 100 – *c_i_* + 0.8Σ*c_j_*	224	12	16–20	24	pair	1	disincent. strategic	0, last
BAYE16c	PG: 100 – *c_i_* + 0.8Σ*c_j_*	224	12	16–20	24	pair	1	incent. strategic	0, last
KAME17a	PG: 10 – *c_i_* + 0.65Σ*c_j_*	120	12	10	40	pair	1	strategic	0, part, avg
KAME17b	PG: 10 – *c_i_* + 0.85	130	13	10	40	pair	1	strategic	0, part, avg
HARR18a	tPD: 50, −50, 100, 0	334	20	12–24	12	(random)	(4)	part strategic	0, avg
HARR18b	tPD: 50, −50, 100, 0	334	20	12–24	12	(random)	(4)	strategic	0, avg
MELA18a	PD: 50, −50, 100, 0	810	15	19–28	16	(random)	(4)	strategic	0, loc, avg
MELA18b	PD: 50, −50, 100, 0	810	15	19–28	16	(clustered)	(4)	strategic	0, loc, avg
MELA18c	tPD: 50, −50, 100, 0	810	15	19–28	16	(random)	(4)	strategic	0, loc, avg
MELA18d	tPD: 50, −50, 100, 0	810	15	19–28	16	(clustered)	(4)	strategic	0, loc, avg
MELA18e	PD: 50, −50, 100, 0	472	16	19–28	16	(random)	(4)	slow strategic	0, loc, avg
MELA18f	tPD: 50, −50, 100, 0	472	14	19–28	16	(random)	(4)	slow strategic	0, loc, avg

My choice to re-analyse existing data, instead of developing bespoke experiments, has some advantages, as well as disadvantages. On the positive side, I insure against Type I errors, or falsely reporting positive effects, because none of the studies could have been cherry-picked to report significant results with respect to the dependent variables. In addition, the repurposed data allow me to affirm the robustness of my findings across different experimental set-ups, incentives, and participant pools; this would be prohibitively expensive to do from scratch. Still, on the negative side, I risk Type II errors, i.e. failing to find true positives, because the experiments were not calibrated to produce the highest variation in the outcome of interest, nor scaled up sufficiently for group-level analyses. I can only test the predictions qualitatively, not quantitatively, because the model and experiments vary in more than one aspect. Finally, I can only do an indirect test of the predictions because only one experiment crosses reputational information with random rewiring and strategic updating. Consequently, I analyse the effect of reputation separately in randomly rewired and strategically updated networks.

The experiments use one of four common social dilemma games: the Trust (TG), Helping (HG), Prisoner's Dilemma (PD) or Public Good (PG) game. The TG is most intuitively understood in the context of buyer­–seller relationships, where the buyer (trustor) decides whether to send a sum of money to the seller (trustee), and if so, the seller gets to choose whether to ship the purchased item, i.e. send something of value back, or not. Honoured trust (*CC*) is mutually beneficial but abusing trust (*CD*) is tempting for the trustee. In the HG, the player decides whether to give a gift of a cost *c* to another player who will benefit *b*, where giving is collectively beneficial (*b* *>* *c*) but not guaranteed to be reciprocated. In the PG, players invest in a common pot and then share the multiplied investments equally. In the PD, players choose either to cooperate or to defect, where mutual cooperation (*CC*) is collectively beneficial but individually unprofitable because defecting (*DC* or *DD*) is always the better choice, regardless of what the other player does.

For random rewiring, I analyse six different network cooperation experiments from five studies [21–23,32,33]. The experiments randomly re-match pairs of participants at regular intervals in networks of 14–24. For strategic updating, I analyse 13 experimental set-ups from five studies [20,25,26,33,34]. Typically, the interaction starts in a network with a certain structure and density, and throughout the game, participants are given the opportunity to drop some of their current interaction partners and select new ones. In the experiments using the PD, participants play the dyadic game with each of their partners; thus, each participant's payoff depends on the number of partners, as well as their actions. In some of these experiments, the participant chooses one action and plays it against all their partners, while in others, they can choose a different action against each partner (shown as tPD, or targeted PD, in table 1). The one experiment that manipulates both reputational information and network updating [33] uses a dyadic PG where partners are updated (randomly assigned or strategically chosen in the different treatments) every four periods.

The experiments manipulate the amount of information available about other players' past actions beyond knowledge of what one's partners did in the previous round. In the control condition, no such information is available and, in [25], even information about what one's own partners individually chose in the previous period is missing. To provide reputational information, some of the experiments reveal to participants what their current or potential partners did in the past *r* periods, where *r* = 1, 3, 5, 6, or all previous periods. Other experiments show the average rate of cooperation/contribution over all previous periods (avg), over half of previous periods selected at random (part avg), or over the last four-period game phase (last avg). One study shows the average rate of cooperation over all previous periods but only towards one's partners' partners (loc avg). Another study includes information on the action of the partner's partner to which one's partner responds (abbreviated as 1 + 1).

For the analyses, I measure final payoff with the number of in-game monetary units the player accumulated at the end of the game. As in the model, I use the Gini coefficient to measure the dispersion of final payoffs. The Gini coefficient is particularly suited in this case because it is invariant to scale, which helps in comparing outcomes across experiments with very different incentive structures [35]. Analogously, I use the Pearson correlation coefficient between the rate of cooperation and final payoff to measure the correspondence between behaviour and rewards.

To test the effects of reputational information on inequality, I compare the Gini and Pearson correlation coefficients in the conditions with reputational information with the baseline condition without. I use the Mann–Whitney test to assess the differences in each control–treatment pair. This is a non-parametric test that does not assume a normal distribution for the residuals. It essentially checks against the null hypothesis that a randomly selected value from one condition would be equally likely to be less than or greater than a randomly selected value from the other condition.

Since the experiments were not designed to test group-level hypotheses, effect sizes and statistical power are not always large enough to provide evidence at the level of a single experiment. Hence, I additionally conduct a meta-analysis across all control–treatment pairs using the sign test [36]. I first count the number of positive and negative effects, regardless of whether they are statistically significant. I then conduct the binomial test, testing against the null hypothesis that there is no effect in reality and thus negative and positive effects are equally likely to occur by chance. This approach is somewhat limited because it does not take into account the amount of evidence: neither the effect magnitudes nor the sample sizes. However, the goal is not to estimate an effect size but to test a causal relationship. Note that effect sizes are not very meaningful in controlled social experiments as they are highly sensitive to aspects of the experimental design such as the framing of the decision situation, the monetary incentives, the experience of the participant pool, and experimenter demand effects [37].

## 5. Results

**Figure 2.**
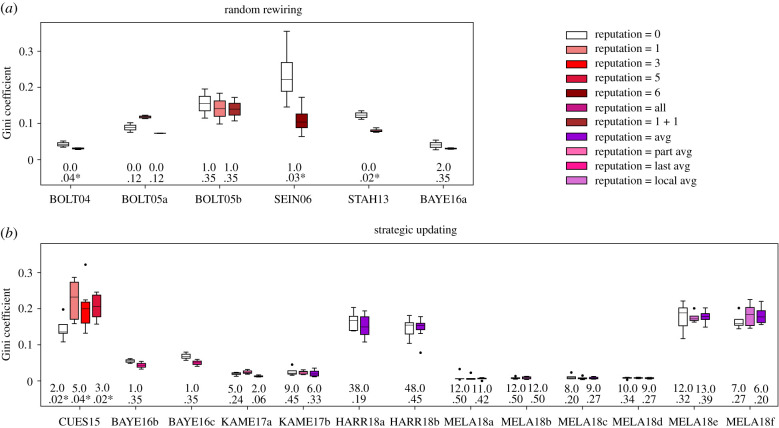
(*a*) The empirical analyses confirm that reputational information decreases the dispersion of final payoffs in randomly rewired networks. (*b*) As predicted, in networks with strategic updating, reputational information could increase the dispersion of final payoffs, as in CUES15, but this does not occur in most cases. The figure shows boxplots of the Gini coefficient for final payoffs for each experimental condition and results from Mann–Whitney tests comparing each condition with reputation with the control condition (reputation = 0) in each experiment (Mann–Whitney *U* on top and 2-sided *p*-value on bottom, with asterisk if *p* < 0.05). Description of the experimental set-ups and treatment conditions can be found in table 1.

I first test the prediction that reputational information lowers the dispersion of payoffs in randomly rewired networks. Using Mann–Whitney tests to compare the Gini coefficients for payoffs accumulated at the end of interactions between the control and treatment conditions, I find statistically significant evidence for this prediction in BOLT04, SEIN06 and STAH13 (figure 2*a*). Overall, the effect direction is as predicted in seven out of the eight control–treatment pairs, yielding a sign test result that is significant at the 0.05-level (1-sided *p* = 0.035).

Next, I investigate whether reputational information could increase the dispersion of payoffs in strategically updated networks. I find statistically significant supporting evidence only in CUES15 (figure 2*b*). Out of the 23 control–treatment pairs, only ten have a positive effect from reputation on inequality, resulting in 2-sided *p* = 0.678 for the sign test. It is worth noting that this result is skewed by eight of the pairs from MELA18 where groups achieve nearly universal cooperation within a couple of periods and, consequently, end up with little variation in final payoffs (electronic supplementary material, figure S4). Nevertheless, even after excluding these outliers, there remain results in both directions (8 positive out of 13, 2-sided *p* = 0.999). In sum, although possible, reputation is unlikely to produce higher inequality in strategically updated networks. This finding corroborates previous research showing that reputation does not contribute much to the clustering and proliferation of cooperators beyond what the possibility to exclude defecting partners can already do [26]. The positive effect found in CUES15 is likely contingent on two design decisions in the experiment: (1) the assumption that memory is a source of reputation, such that in the no reputation condition, individuals do not know even their own partners' actions, and (2) the payoff matrix with *CD* = *DD*, which means that cooperators do not lose by interacting with defectors. Because of these two assumptions, exclusion does not occur when reputation = 0, evidenced by the fact that the networks have much higher density then [25].

**Figure 3.**
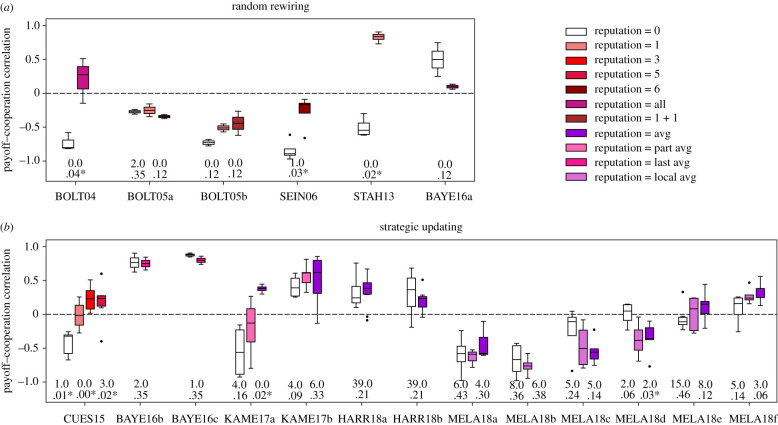
Reputational information generally improves the correspondence of payoffs to cooperation. The overall effect, however, is only significant in strategically updated networks (*b*) if MELAa-d is excluded, where the level of cooperation is greater than 95%. The figure shows boxplots of the Pearson correlation between final payoffs and individual cooperation, the latter defined as the proportion of periods in which the participant chose to cooperate. The text shows results from Mann–Whitney tests comparing each condition with reputation with the control condition (reputation = 0) in each experiment (Mann–Whitney *U* on top and 2-sided *p*-value on bottom, with asterisk if *p* < 0.05).

Regarding the expected positive effect of reputational information on the correspondence between payoffs and cooperative behaviour, I find supporting evidence that is statistically significant in BOLT04, SEIN06, STAH13, CUES15 and KAME17a (figure 3). The overall results, however, are not statistically significant for randomly rewired networks (6/8, 1-sided *p* = 0.145) and significant for strategically updated networks only if the eight outliers from MELA18 are removed (12/15, 1-sided *p* = 0.018). Figures S5 and S6 in the electronic supplementary material show in more detail how reputational information increases both the level of cooperation and the rewards for cooperating in each experiment.

## 6. Discussion

This study aimed to expand our understanding of the effects of reputation systems in social groups. Complementing prior research, which focuses on cooperation and collective welfare, I investigated how reputational information affects inequality. I studied inequality in terms of both the dispersion and fairness of rewards: I analysed how the final payoffs of the poorest differ from those of the richest, but also how the final payoffs correspond to cooperative behaviour. Overall, my aim was to answer the question of whether reputation systems pose a tradeoff between efficiency and equality: Do the higher levels of cooperation and higher collective wealth imply undesirable side effects that have been overlooked? I argued and demonstrated with an agent-based model that reputation may increase the dispersion of payoffs under some conditions in strategically updated networks, but almost always decreases it in randomly rewired networks; it also provides better rewards to cooperative behaviour regardless of the dynamics of the interaction network.

I hypothesized and found partial empirical evidence that reputational information decreases inequality in interaction situations where partners are periodically randomly re-matched. Markets driven by one-off transactions that employ some form of randomization in allocating sellers to buyers exemplify such situations. Ride-hailing services such as Uber and Lyft present a good case in point, as they match drivers with customers based on time availability, which introduces a component of randomness. Networks that are periodically randomly rewired can also occur in schools and work environments, when teachers and managers randomly allocate students or employees to team projects. In this kind of networks, reputation increases the level of cooperation but also the rewards for cooperating, and this diminishes the dispersion of final payoffs. In other words, in randomly rewired networks, reputation works even better than expected: it benefits both efficiency and equality.

In interaction situations where individuals strategically choose their partners, I predicted that reputational information increases the dispersion of payoffs only under restricted conditions and, indeed, I found that this is not common empirically. More importantly, just as in randomly rewired networks, in networks with strategic updating, reputation increases the rewards to cooperators and any increase in the dispersion of payoffs occurs at the expense of defectors, who are excluded and isolated. Yet, in practice, as [26] argues, strategic updating can already induce segregation between cooperators and defectors, and reputation does not contribute additionally. Thus, even in situations where partners are strategically selected, reputation systems appear to improve equality and fairness.

This study extends and qualifies previous research showing that reputation systems could produce arbitrary and enduring inequalities in social dilemma situations [16,17]. Its first contribution is to introduce, in addition to the dispersion of payoffs, another dimension to consider when analysing inequality in cooperation settings—the *correspondence* between payoffs and cooperative behaviour. This dimension is important to the extent to which cooperative behaviour is attributed to structural constraints and incentives, rather than individual agency. If cooperation is enabled by an arbitrary resource advantage [16] or constrained by the lack of opportunities to act [17], correspondence is less meaningful. When everyone faces the same decision situation, however, our sense of justice and fairness tells us that rewards should correspond to actions and this becomes an important consideration for inequality.

The second contribution to existing research is to account for the multiplicity of equilibria. I accomplished this by presenting a generic model that considers different combinations of individual strategies and analysing experiments with different incentives and set-ups. This approach does not allow explanation of differences in the results between the model and any specific experiment, or between any pair of experiments, owing to the fact that the model and the experiments differ on more than one dimension. Nevertheless, the approach theoretically allows isolation of the main driving factors behind the phenomenon I studied—the emergent level of behaviour, the variability in individual behaviour, and the assortativity between individuals with similar behaviour—and testing of the frequency and robustness of the predictions under variable empirical settings. This work brings attention to the danger of drawing strong conclusions from a single model or experiment. Specifically, although prior research offers empirical evidence for the intuitive expectation that strategic partner selection increases inequality in terms of payoff dispersion [17], I have revealed that this outcome is in fact rare for most network cooperation settings.

I acknowledge that the results presented here are not conclusive and that further research is needed. The biggest limitation of this study is that the empirical results regarding strategically updated networks are based only on symmetrical games, where both parties choose partners and face the same decision situation. What is more, the networks are degree-constrained, such that most individuals have a similar number of interaction partners. For example, this is the case when students team up for a course project. By contrast, buyer–seller markets involve asymmetric relations and preferential attachment, and research of such settings suggests that reputation systems could produce enduring inequalities [17,38,39]. This study could be extended with a bespoke experiment that investigates the effects of reputational information on inequality in strategically updated networks for both symmetric and asymmetric social dilemma games, systematically varying the maximum number of possible partners. This would test whether the negative effects of reputation on inequality are contingent on preferential attachment and exclusion, which prevent the opportunity to modify behaviour.

Overall, the present finding that reputation systems rarely worsen inequality in social dilemma situations in degree-constrained networks and, in fact, improve it under most conditions and in terms of rewarding cooperative behaviour suggests that it is possible to benefit from reputation systems without undesirable side effects. If reputation is used to encourage defectors to cooperate, rather than punish and isolate them, then everyone will be better off. This could be achieved by, for instance, implementing smart search algorithms in online markets and more deliberate team-formation strategies in schools and organizations.

## Data accessibility

The processed data, NetLogo scripts for the model, and Python scripts for the analyses are available at: https://doi.org/10.6084/m9.figshare.15097107 [28].

## References

[1] Roberts G, Raihani N, Bshary R, Manrique HM, Farina A, Samu F, Barclay P. 2021 The benefits of being seen to help others: indirect reciprocity and reputation-based partner choice. Phil. Trans. R. Soc. B **376**, 20200290. (10.1098/rstb.2020.0290) 34601903 PMC8487748

[2] Nowak MA, Sigmund K. 2005 Evolution of indirect reciprocity. Nature **437**, 1291-1298. (10.1038/nature04131) 16251955

[3] Wedekind C, Milinski M. 2000 Cooperation through image scoring in humans. Science **288**, 850-852. (10.1126/science.288.5467.850) 10797005

[4] Milinski M, Semmann D, Krambeck H-J. 2002 Reputation helps solve the ‘tragedy of the commons’. Nature **415**, 424-426. (10.1038/415424a) 11807552

[5] Fu F, Hauert C, Nowak MA, Wang L. 2008 Reputation-based partner choice promotes cooperation in social networks. Phys. Rev. E **78**, 026117. (10.1103/physreve.78.026117) PMC2699261 18850907

[6] Wu J, Balliet D, Lange PAMV. 2016 Reputation, gossip, and human cooperation. Social Personal. Psychol. Compass **10**, 350-364. (10.1111/spc3.12255)

[7] Stainback K, Tomaskovic-Devey D, Skaggs S. 2010 Organizational approaches to inequality: inertia, relative power, and environments. Annu. Rev. Sociol. **36**, 225-247. (10.1146/annurev-soc-070308-120014)

[8] Bapuji H, Ertug G, Shaw JD. 2019 Organizations and societal economic inequality: a review and way forward. Acad. Manag. Ann. **14**, 60-91. (10.5465/annals.2018.0029)

[9] Alesina A, Di Tella R, MacCulloch R. 2004 Inequality and happiness: are Europeans and Americans different? J. Public Econ. **88**, 2009-2042. (10.1016/j.jpubeco.2003.07.006)

[10] Luttmer EFP. 2005 Neighbors as negatives: relative earnings and well-being. Q. J. Econ. **120**, 963-1002. (10.1093/qje/120.3.963)

[11] Wilkinson R, Pickett K. 2010 The spirit level: why equality is better for everyone. London, UK: Penguin.

[12] Takács K, Gross J, Testori M, Letina S, Kenny AR, Power EA, Wittek RPM. 2021 Networks of reliable reputations and cooperation: a review. Phil. Trans. R. Soc B **376**, 20200297. (10.1098/rstb.2020.0287) 34601917 PMC8487750

[13] Merton RK. 1988 The Matthew effect in science, II: cumulative advantage and the symbolism of intellectual property. Isis **79**, 606-623. (10.1086/354848)

[14] DiPrete TA, Eirich GM. 2006 Cumulative advantage as a mechanism for inequality: a review of theoretical and empirical developments. Annu. Rev. Sociol. **32**, 271-297. (10.1146/annurev.soc.32.061604.123127)

[15] Petersen AM, Jung W-S, Yang J-S, Stanley HE. 2011 Quantitative and empirical demonstration of the Matthew effect in a study of career longevity. Proc. Natl Acad. Sci. USA **108**, 18-23. (10.1073/pnas.1016733108) 21173276 PMC3017158

[16] Hackel LM, Zaki J. 2018 Propagation of economic inequality through reciprocity and reputation. Psychol. Sci. **29**, 604-613. (10.1177/0956797617741720) 29474134

[17] Frey V, van de Rijt A. 2016 Arbitrary inequality in reputation systems. Scient. Rep. **6**, 38304. (10.1038/srep38304) PMC5171920 27995957

[18] Lynn FB, Podolny JM, Tao L. 2009 A sociological (de)construction of the relationship between status and quality. Am. J. Sociol. **115**, 755-804. (10.1086/603537)

[19] Gallo E, Yan C. 2015 The effects of reputational and social knowledge on cooperation. Proc. Natl Acad. Sci. USA **112**, 3647-3652. (10.1073/pnas.1415883112) 25775544 PMC4378402

[20] Kamei K, Putterman L. 2017 Play it again: partner choice, reputation building and learning from finitely repeated dilemma games. Econ. J. **127**, 1069-1095. (10.1111/ecoj.12320)

[21] Bolton GE, Katok E, Ockenfels A. 2004 How effective are electronic reputation mechanisms? An experimental investigation. Manag. Sci. **50**, 1587-1602. (10.1287/mnsc.1030.0199)

[22] Bolton GE, Katok E, Ockenfels A. 2005 Cooperation among strangers with limited information about reputation. J. Public Econ. **89**, 1457-1468. (10.1016/j.jpubeco.2004.03.008)

[23] Seinen I, Schram A. 2006 Social status and group norms: indirect reciprocity in a repeated helping experiment. Eur. Econ. Rev. **50**, 581-602. (10.1016/j.euroecorev.2004.10.005)

[24] Corten R, Rosenkranz S, Buskens V, Cook KS. 2016 Reputation effects in social networks do not promote cooperation: an experimental test of the Raub & Weesie model. PLoS ONE **11**, e0155703. (10.1371/journal.pone.0155703) 27366907 PMC4930174

[25] Cuesta JA, Gracia-Lázaro C, Ferrer A, Moreno Y, Sánchez A. Reputation drives cooperative behaviour and network formation in human groups. Scient. Rep. **5**, 7843. (10.1038/srep07843) PMC4297950 25598347

[26] Melamed D, Harrell A, Simpson B. 2018 Cooperation, clustering, and assortative mixing in dynamic networks. Proc. Natl Acad. Sci. USA **115**, 951-956. (10.1073/pnas.1715357115) 29339478 PMC5798352

[27] Tsvetkova M, Wagner C, Mao A. 2018 The emergence of inequality in social groups: network structure and institutions affect the distribution of earnings in cooperation games. PLoS ONE **13**, e0200965. (10.1371/journal.pone.0200965) 30028884 PMC6054378

[28] Tsvetkova M. 2021 The effects of reputation on inequality in network cooperation games. Figshare. (10.6084/m9.figshare.15097107) PMC8487746 34601921

[29] Fischbacher U, Gächter S, Fehr E. 2001 Are people conditionally cooperative? Evidence from a public goods experiment. Econ. Lett. **71**, 397-404. (10.1016/S0165-1765(01)00394-9)

[30] Kurzban R, Houser D. 2005 Experiments investigating cooperative types in humans: a complement to evolutionary theory and simulations. Proc. Natl Acad. Sci. USA **102**, 1803-1807. (10.1073/pnas.0408759102) 15665099 PMC547861

[31] Spadaro G, Tiddi I, Columbus S, Jin S, ten Teije A, Balliet D. 2020 *The Cooperation Databank*. See 10.31234/osf.io/rveh3 (accessed 12 May 2021). PMC9442633 35580271

[32] Stahl DO. 2013 An experimental test of the efficacy of a simple reputation mechanism to solve social dilemmas. J. Econ. Behav. Org. **94**, 116-124. (10.1016/j.jebo.2013.08.014)

[33] Bayer R-C. 2016 Cooperation in partnerships: the role of breakups and reputation. J. Inst. Theor. Econ. **172**, 615-638. (10.1628/093245616X14610627109836)

[34] Harrell A, Melamed D, Simpson B. 2018 The strength of dynamic ties: the ability to alter some ties promotes cooperation in those that cannot be altered. Sci. Adv. **4**, eaau9109. (10.1126/sciadv.aau9109) 30525106 PMC6281432

[35] Allison PD. 1978 Measures of inequality. Am. Sociol. Rev. **43**, 865-880. (10.2307/2094626)

[36] Borenstein M. 2009 Introduction to meta-analysis, 1st edn. Chichester, UK: John Wiley & Sons.

[37] Zizzo DJ. 2010 Experimenter demand effects in economic experiments. Exp. Econ. **13**, 75-98. (10.1007/s10683-009-9230-z)

[38] van de Rijt A, Kang SM, Restivo M, Patil A. 2014 Field experiments of success-breeds-success dynamics. Proc. Natl Acad. Sci. USA **111**, 6934-6939. (10.1073/pnas.1316836111) 24778230 PMC4024896

[39] Salganik MJ, Dodds PS, Watts DJ. 2006 Experimental study of inequality and unpredictability in an artificial cultural market. Science **311**, 854-856. (10.1126/science.1121066) 16469928

